# What models of community palliative rehabilitation exist for adults in the United Kingdom? – a national cross-sectional survey

**DOI:** 10.1186/s12904-026-02027-x

**Published:** 2026-03-13

**Authors:** Jane Manson, Paul Taylor, Susan Mawson, Alicia O’Cathain

**Affiliations:** 1 Sheffield Teaching Hospitals NHS Foundation Trust, Firth Park Clinic, 16 Sicey Avenue, Sheffield, S5 0RR UK; 2 Sheffield Centre for Health and Related Research, The University of Sheffield, Sheffield, UK; 3 The University of Sheffield, Sheffield, UK; 4 St Luke’s Hospice, Sheffield, UK

**Keywords:** Rehabilitation, Rehabilitative Palliative Care, Models, Community

## Abstract

**Background:**

Palliative care for advanced illness is expected to increase globally by 87% by 2060. Palliative rehabilitation helps patients manage symptoms and maintain independence. It is increasingly delivered in community settings where practitioners visit patients at home, or in outpatient clinics. However, there is limited current evidence on how community palliative rehabilitation is delivered in the UK.

**Aim:**

To describe current models of community palliative rehabilitation provision.

**Methods:**

A cross-sectional online survey aimed at senior clinicians or managers of adult hospices that outreach into the community and generalist community palliative rehabilitation services in the United Kingdom in 2024.

**Results:**

Of the 381 NHS community rehabilitation and specialist palliative care organisations surveyed, 96 (25%) responded, with most being independently funded hospices or nationally funded hospital trusts (NHS Trusts). Partial responses were included. All organisations employed physiotherapists and most (96%, 74/77) employed occupational therapists in their community palliative rehabilitation teams but over half did not employ any other AHPs (60%, 46/77). Independent hospices were more likely to employ specialist palliative care physiotherapists (71% vs. 21%) and occupational therapists (54% vs. 30%) than NHS community organisations and treat patients with cancer and COPD (100% vs. 68% respectively for cancer, 100% vs. 59% respectively for COPD). Most clinicians (89%), irrespective of organisation type, asked for further education or guidance to treat patients with palliative diagnoses. Many organisations could not always provide information in languages other than English (86% 57/66) or interpretation services at appointments (51%, 34/66). Integration of community rehabilitation services was better with specialist palliative care services (50%, 30/60) and primary care (42%, 26/60), but weaker with secondary care (21%, 13/60) and third sector organizations (11%, 7/60).

**Conclusions:**

There is variation in community palliative rehabilitation models across the United Kingdom in terms of staffing composition, casemix, equity of access for patients, and integration with other services. Further education or guidance is needed for clinicians in rehabilitation teams supporting patients with palliative diagnoses.

**Supplementary Information:**

The online version contains supplementary material available at 10.1186/s12904-026-02027-x.

## Background

Palliative care is defined by the National Institute for Health and Care Excellence as: “The active holistic care of patients with advanced, progressive illness” [1]. International guidance proposes that the need for palliative care will increase up to 87% globally by 2060 [2]. Palliative rehabilitation is an important part of palliative care. The World Health Organisation states that palliative rehabilitation “empowers people with incurable health conditions to actively manage their condition, reduces symptoms and enables individuals to stay independent and socially active” [3]. This could reduce the burden on international health systems and improve patients’ quality of life.

There has been a distinction in the literature between “palliative rehabilitation” and “rehabilitative palliative care” [4]. The former is when rehabilitation is carried out by trained specialists such as physiotherapists and occupational therapists. The latter relies on a whole team approach to create a culture of rehabilitation where optimising function is a key goal of care. While some data on numbers of wider Allied Health Professionals (AHPs) have been included for interest, the survey was mainly focussed on palliative rehabilitation.

Palliative rehabilitation can be delivered within inpatient care or in the community where practitioners visit patients in their own homes. The latter is called community palliative rehabilitation. There is an increasing push for care to be delivered closer to home in the United Kingdom (UK) [5], and globally [6], including rehabilitation [7]. Community rehabilitation is distinct from rehabilitation delivered in an inpatient hospital or hospice setting and can reach patients that are unable to access these services due to transport, anxiety or social issues. There is little evidence on how community palliative care rehabilitation is delivered. A survey by Wosahlo and Maddocks [8] in 2015 investigated models of hospice-based palliative rehabilitation in the UK. However, it is dated and focused on independent hospices rather than palliative rehabilitation provided by the breadth of services including hospices, the National Health Service (NHS) and/or non-hospice community providers and did not focus only on community palliative rehabilitation. A recent realist review has aimed to identify aspects of physiotherapy and occupational therapy in palliative rehabilitation that could include outcomes [9], however it is not known what currently happens in practice in the community in the UK.

The recent James Lind Alliance priorities in palliative and end of life care highlight a need for further research into supporting patients with a palliative diagnosis in the community [10]. This study sought to investigate current models of community palliative rehabilitation delivery by a variety of services in the UK, to understand what models exist and to inform future research and clinical practice in this area.

## Methods

### Setting

This is a UK-based study of providers of community palliative rehabilitation. In the UK, community palliative rehabilitation is either provided by independent hospices which are predominantly funded by charities, or by NHS Foundation or Community Trusts that are predominantly funded by the government, and which may include an NHS hospice or palliative care service. Provision is largely for adults, with palliative care for children provided by specialist children’s hospices or children’s NHS services. Both independent hospices and NHS trusts were targeted for the survey if they had rehabilitation services that outreached into the community.

### Study design

The study was a cross-sectional online survey aimed at senior clinicians or managers of independent hospices or NHS generalist community rehabilitation services in the UK in 2024. The focus was on adults because adult and paediatric hospice services tend to be organised and function differently despite having similar aims [11].

### Sample

The target population was the 190 UK adult independent hospices and 191 NHS organisations which deliver adult community palliative rehabilitation. A database of contact email addresses was developed from publicly available information. This involved ascertaining a list of UK adult independent hospices [12] and NHS Trusts [13] and searching for rehabilitation or general contact details. It was not always possible from this information to ascertain if organisations delivered community palliative rehabilitation. Therefore, the cover letter and first question on the questionnaire made it clear that the survey was only to be completed about community palliative rehabilitation. Trusts that did not deliver palliative rehabilitation to patients in the community were thanked for their input with no further questions posed.

### The questionnaire

As there are no existing valid or reliable tools in this area, the questionnaire was designed based on two sources. First, several questions from the Wosahlo & Maddocks survey were used with permission [8]. Second, further questions were based on a realist review exploring aspects of models of community palliative rehabilitation that could improve outcomes for patients [14]. These explored team composition, integration, access to services, and education.

The questionnaire was developed by the lead researcher alongside a group consisting of academics, clinicians, a service manager, and patient representatives (see supplementary material for final questionnaire). A definition of clinical specialist was added following consultation with this group, and extra questions were included to further understand access to community palliative rehabilitation. The questionnaire was pilot tested with five further individuals with no extra changes needed. The questionnaire was delivered using Qualtrics© [15] and was available only in English as it was assumed that all senior clinicians or managers completing it would have a good understanding of English.

The final questionnaire contained 36 questions within 11 blocks including multiple choice, matrix, Likert scales, and short answer questions. Participants saw a maximum of four questions per page over 18 pages and items were not randomised. Information was presented in the cover letter/introductory email with a further explanation embedded in the first page of the questionnaire. Participants were able to review and change their answers prior to their final submission using the back button and they were also able to leave the survey and return to their saved answers until data collection was closed.

### Ethical approval and consent to participate

Ethical approval was received by the Health Research Authority and Health Care Research Wales under proportionate review in May 2024 (IRAS 342949) and data collection took place for eight weeks July-September 2024. All data collection adhered to the ethical principles outlined in the Declaration of Helsinki [16].

A participation sheet was embedded within the cover letter. Informed consent was implied once participants read the cover letter and clicked on the questionnaire to continue. The final page of the questionnaire included participant consent to be contacted for further research on this topic. No incentives were offered to participants for taking part. Participants were able to review and change their answers prior to their final submission.

### Data collection

A link to the online questionnaire was distributed to the sample via email from the central platform. Only individuals who had access to the survey link were able to access the survey and CAPTCHA was used to filter out automated responses. Unique IP addresses were collected to ensure the survey was only completed once per organisation. Special interest groups and social media were also utilised to advertise the research. Completion of the questionnaire was voluntary. A reminder to complete questions left blank before progressing was included, but users could override this if they did not wish to answer any question. Responses to the questionnaire were exported to SPSS for analysis. Data was anonymised and kept securely in a password protected folder within an encrypted database.

### Analysis

Responses to the questionnaire were collected automatically on Qualtrics© [15] and exported to SPSS for analysis. A respondent was defined as a service identified by a unique IP address that had completed at least one question.

Descriptive statistics were produced for each question. Different aspects of models of care were compared by independent hospice vs. NHS services and chi-squared tests undertaken. For analysis purposes, independent hospices were defined as charity funded, and NHS services included hospices and other services which are primarily NHS funded. For some variables response categories were collapsed to ensure the chi-squared tests were valid.

## Results

### Response rate

Ninety-six of 381 (25%) organisations completed all or part of the questionnaire. 57/381 (15%) completed all of it. The most frequent drop-out point was at question 16 of 36. Services with partial responses were included in the analysis therefore some questions may have more responses than others. There were no duplicate entries.

### Characteristics of organisations

The distribution of organisation type in the sample was representative of the UK distribution of independent hospices and NHS trusts in terms of location (National: England 87%, Northern Ireland 3%, Scotland 6%, Wales 4%). See Table 1. Most organisations were independent hospices or NHS Trusts. The modal population size covered was 250,000 to 500,000. Most organisations covered urban, or mixed urban and rural, areas.

**Table 1:** Characteristics of organisations responding

Characteristic	Categories	*N*	(%)
Location	England	80	(84.2)
	Northern Ireland	2	( 2.1)
	Scotland	12	(12.6)
	Wales	1	(1.1)
Type of organisation	Independent hospice	32	(33.3)
	NHS hospice	1	(1)
	NHS palliative care unit	2	(2.1)
	NHS Trust	57	(59.4)
	Other (NHS & Charity Partnership Hospice x 2 Health and Social Care x 1 Blank x 1)	4	(4.2)
Population size organisation covers	Under 50,000	5	(5.3)
	50,000-100,000	8	(8.5)
	100,000-250,000	24	(25.5)
	250,000-500,000	28	(29.8)
	500,000-750,000	12	(12.8)
	750,000–1,000,000	9	(9.6)
	Over 1,000,000	8	(8.5)
Population density	Urban	27	(28.1)
	Rural	8	(8.4)
	Mixed urban and rural	56	(58.3)
	Island	4	(4.2)
	Other (not stated)	1	(1)
Percentage of patients seen that are palliative	Less than 10%	11	(15.3)
	10–25%	15	(20.8)
	25–50%	5	(6.9)
	50–75%	4	(5.6)
	Over 75%	37	(51.4)

Partial survey responses included; % reflects proportion of completed responses

### Types of patients in palliative caseload

Most services had a majority palliative caseload, although 15.3% of respondents reported patients with a palliative diagnosis taking up less than 10% of their total patients seen (Table 1). People with cancer were the most common patient group followed by those with chronic obstructive pulmonary disease (COPD) and advanced frailty (Table 2). Some services reported never managing people with COPD or cancer, indicating the specialist nature of some services. Interestingly dementia and advanced frailty were the only diagnoses managed across all services (Table 2).

**Table 2:** Frequency of managing different types of patients in palliative caseload

	Very frequently/ daily		Frequently/ weekly		Occasionally/ monthly		Rarely/few times per year		Never	
	*N*	%	*N*	%	*N*	%	*N*	%	*N*	%
Cancer	45	62.5	14	19.4	9	12.5	2	2.8	2	2.8
COPD	23	31.9	32	44.4	11	15.3	1	1.4	5	7
Heart Failure	9	12.5	35	48.6	22	30.6	2	2.8	4	5.5
Parkinson’s and related conditions	11	15.3	20	27.8	29	40.3	10	13.9	2	2.7
Dementia	15	21.1	22	31.0	23	32.4	11	15.5	0	0
Advanced Frailty	23	31.9	27	37.5	14	19.5	8	11.1	0	0
End-stage kidney disease	1	1.4	14	20.0	37	52.9	16	22.9	2	2.8
End stage liver disease	1	1.4	9	12.9	33	47.1	24	34.3	3	4.3
Motor Neurone Disease	7	9.9	15	21.1	26	36.6	19	26.8	4	5.6
Other progressive neurological conditions	4	5.6	19	26.8	28	39.4	19	26.8	1	1.4

Partial survey responses included; % reflects proportion of completed responses

The frequency of each type of patient was compared by service type. To do this, NHS services were grouped together and compared with independent hospices and “other” services (see Table 1 for types of organisation). There was a statistically significant difference for cancer and COPD: these patients were managed very frequently and frequently by independent hospice services compared with NHS Services (cancer 100% vs. 68%, *X*²=10.94, d.f.=1, *N* = 69, *p* < 0.001, Cramer’s V = 0.398 – medium effect size, COPD 100% vs. 59%, *X*²15.41, d.f.=1, *N* = 69, *p* < 0.001, Cramer’s = V 0.473 – medium effect size) whereas Parkinson’s Disease and related conditions and patients with a dementia diagnosis were more likely to be managed very frequently and frequently by NHS services compared with independent hospices (Parkinsons 25% vs. 56%, *X*²=6.55, d.f.=1, *N* = 69, *p* = 0.011, Cramer’s V = 0.308 – medium effect size and dementia 30% vs. 71%, *X*²=11.09, d.f.=1,*N* = 68, *p* < 0.001, Cramer’s V = 0.404 – medium effect size respectively).

### Types of AHPs providing care

Table 3 shows the number of AHPs employed by organisations in their community rehabilitation services. All organisations employed physiotherapists, and all bar three employed occupational therapists. Apart from physiotherapists and occupational therapists, over 60% of organisations did not employ other AHPs within their community rehabilitation services. Other clinicians highlighted in the free text were complementary therapists (2), therapy assistants (6) and associate practitioners (1).

**Table 3:** Different types of AHPs Employed

	0		Less than 1 FTE		1 to 2 FTE		3 to 5 FTE		6 to 10 FTE		11 to 20 FTE		Over 20 FTE	
	*N*	%	*N*	%	*N*	%	*N*	%	*N*	%	*N*	%	*N*	%
Art Therapists	32	76.2	6	14.3	4	9.5	0	0	0	0	0	0	0	0
Dieticians	28	65.1	7	16.3	2	4.7	3	7.0	1	2.3	1	2.3	1	2.3
Drama Therapists	34	91.9	2	5.4	1	2.7	0	0	0	0	0	0	0	0
Lymphoedema Specialists	27	65.9	5	12.2	7	17.1	1	2.4	0	0	0	0	1	2.4
Music Therapists	30	30	81.1	4	10.8	2	5.4	0	0	0	0	0	1	2.4
Occupational Therapists	3	3.9	13	16.9	22	28.5	18	23.4	7	9.1	6	7.8	8	10.4
Orthotists	32	84.4	1	2.6	1	2.6	2	5.2	0	0	1	2.6	1	2.6
Paramedics	31	79.5	2	5.1	1	2.6	1	2.6	2	5.1	0	0	1	2.6
Physiotherapists	0	0	8	10.4	24	31.1	17	22.1	13	16.9	6	7.8	9	11.7
Podiatrists	27	69.1	4	10.3	3	7.7	1	2.6	1	2.6	2	5.1	1	2.6
Speech and Language Therapists	25	62.5	3	7.5	1	2.5	6	15	2	5	2	5	1	2.5

Partial survey responses included; % reflects proportion of completed responses

### Specialist palliative rehabilitation

The definition of clinical specialist specified in the questionnaire was “a clinician who has undergone specialist palliative care training and in the NHS is usually band 7 or above”. Thirty-two (43%) of 75 services had at least one palliative care clinical specialist physiotherapist and thirty-three (44%) of 75 services had at least one palliative care clinical specialist occupational therapist. The number of clinical specialist occupational therapists and physiotherapists were compared by service type as above. This showed that independent hospices were more likely to employ clinical specialist physiotherapists (71% vs. 21%, *X*²=14.82, d.f.=1, *N* = 75, *p* < 0.001, Cramer’s V = 0.495 – medium effect size) and occupational therapists (54% vs 30%, *X*²= 6.83, d.f.=1, *N* = 75, *p* = 0.001, Cramer’s V = 0.236 – small effect size) compared with NHS services. There was no statistically significant difference when comparing the number of clinical specialist staff with population size.

### Therapists requiring further education or advice

54/63 (86%) organisations reported that therapists always or usually report that they need further education or advice to provide community rehabilitation to patients with a palliative diagnosis. Therapists working in NHS organisations were more likely to require education or advice than those in hospice services (94% vs. 74%, *X*²=5.28, d.f.=1, *N* = 63, *p* = 0.22, Cramer’s V = 0.358 – moderate effect size).

### Locations of care

The reported locations of care and frequency (%) of provision are provided in Table 4. Participants could provide multiple answers dependent on their service. The most common location of care was rehabilitation in the patient’s own home followed by 1:1 interventions on the organisation’s site. Independent hospice and NHS provision were again compared to see if there was a difference in their delivery of community palliative rehabilitation. This demonstrated that independent hospices were more likely to deliver 1:1 (100% vs. 53%, *X*²=16.92, d.f.=1, *N* = 62, *p* < 0.001, Cramer’s V = 0.522 – large effect size) and group (88% vs. 31%, *X*²=19.60, d.f.=1, *N* = 61, *p* < 0.001, Cramer’s V = 0.567 – large effect size) models on the organisation’s site than NHS organisations. There was no statistically significant difference between independent hospice/NHS and any of the other locations indicating that they are provided in both settings.

**Table 4:** Models of Care

	Person’s own home		1:1 not on organisation site		Group not on organisation site		1:1 on organisation site		Group on organisation site		Telerehabilitation	
	*N*	%	*N*	%	*N*	%	*N*	%	*N*	%	*N*	%
Independent Hospice	22	85	11	44	4	17	26	100	22	88	15	63
NHS	37	97	14	38	8	22	19	53	11	31	14	39
Total	59	92	25	40	12	20	45	73	33	54	29	48

Partial survey responses included; % reflects proportion of completed responses

Organisations can offer more than one service so %s sum to more than 100

### Access requirements

58/69 (84%) services reported that there were eligibility criteria to access palliative rehabilitation. The most common access requirements were for patients to have a palliative diagnosis or symptoms (62%), be referred by a healthcare professional (32%), or to be able to participate in services independently (able to mobilise/toilet self/follow simple instructions) (13%).

Further questions were asked to determine the accessibility of services for patients. Most services were able to offer rehabilitation at a time and location to meet a patient’s needs. However, a barrier to accessing services was where English was not a patient’s first language. Only 14% of services reported to always offer information in languages other than English and less than 50% of services reported always offering interpretation services (Table 5).

Over 84% of organisations felt that the patients that accessed their services were representative of the population in the catchment that they served.

**Table 5:** Accessibility of services for patients

	Always		Usually		Sometimes		Rarely		Never		Don’t know	
	*N*	%	*N*	%	*N*	%	*N*	%	*N*	%	*N*	%
Are you able to offer rehabilitation at a time which suits the patient?	13	19.7	45	68.2	6	9.1	2	3	0	0	0	0
Can you offer different formats or locations to meet someone’s needs (e.g. home-based, outpatient, one-to-one, group-based, virtual)	13	19.7	25	37.9	14	21.2	9	13.6	4	6.1	1	1.5
Do you offer information in languages other than English?	9	13.6	12	18.2	14	21.2	20	30.4	9	13.6	2	3
Are you able to offer interpreter services for patients who do not have English as a first language?	32	48.5	21	31.8	10	15.2	2	3	0	0	1	1.5
Are the patients that access your service representative of your organisations’ catchment area?	22	33.3	34	51.5	8	12.2	1	1.5	0	0	1	1.5

Partial survey responses included; % reflects proportion of completed responses

### Integration with other services

Services were asked about how integration was achieved with other community services such as social care and voluntary services (Table 6). The most common method of communication with services was liaison only (e.g. telephone and letters), followed by joint clinical meetings. There were also some examples of cross-organisational service delivery. Two organisations (one NHS community and one health and social care) reported no contact with their local independent hospice. A further two (one NHS community and one independent hospice) reported no contact with their local social care organisation.

**Table 6:** How integration is achieved with community services

	Responder’s own service		Cross-organisational service delivery e.g. joint funded AHP posts, joint group programmes		Joint clinical meetings e.g. MDT		Liaison only e.g. telephone and letters		No collaboration		Not applicable	
	*N*	%	*N*	%	*N*	%	*N*	%	*N*	%	*N*	%
Community NHS	28	45.2	6	9.6	14	22.6	14	22.6	0	0	0	0
Independent Hospice	20	32.3	4	6.4	11	17.7	20	32.3	2	3.2	5	8.1
Social Care	4	6.5	8	12.9	16	25.8	31	50	2	3.2	1	1.8
Other voluntary organisation	3	4.8	3	4.8	11	17.7	35	56.5	4	6.5	6	9.7
Other	3	4.8	2	3.2	1	1.6	3	4.8	4	6.5	49	79.1

Partial survey responses included; % reflects proportion of completed responses

Other (free text): Acute services 1, Council 1, Any relevant party 1, Other therapy services 1, Oncology nurse specialists 1, Community respiratory services 1

% in brackets

Organisations were also asked about their integration with primary care, secondary care, other voluntary organisations, and specialist palliative care. Half of the organisations who responded reported that they were integrated with specialist palliative care services (50%, 30/60). Slightly less (42%, 26/60) noted that they were well integrated with primary care. Integration occurred less with secondary care and third sector organisations, with only 21% (13/60) (secondary care) and 11% (7/60) (third sector) organisations reported that they were well integrated.

Independent hospices reported better integration with specialist palliative care than NHS services (80% vs. 29%, *X*²=15.43, d.f.=1, *N* = 60, *p* < 0.001, Cramer’s V = 0.507 – large effect size) but there were no other statistically significant differences.

There was a relationship between the level of integration with primary care (*X*²=5.12, d.f.=1, *N* = 60, *p* = 0.028, Cramer’s V = 0.292 – low effect size) and secondary care (*X*²=4.82, d.f.=1, *N* = 60, *p* > 0.028, Cramer’s V = 0.346 – medium effect size) and a perceived understanding of community palliative rehabilitation by other organisations in that the higher the level of perceived integration, the higher the level of perception that other services understood the community palliative rehabilitation service (37% for good integration and good understanding vs. 6% for poor integration and poor understanding in Primary Care and 27% for good integration and good understanding vs. 6% for poor integration and good understanding in Secondary Care).

## Discussion

This research identifies discrepancies between independent hospice and NHS generalist community rehabilitation services with the diagnostic composition of patients. It also identifies a mismatch between the palliative rehabilitation services currently available in the community and those supported by evidence as effective in improving outcomes such as inequitable access, integration with other services, workforce patterns, and educational needs.

### Diagnostic composition of palliative rehabilitation

It is unsurprising that patients with a diagnosis of cancer or COPD were seen more by independent hospice services. The Office for Health and Disparities reported that over 80% of independent hospice patients in England in 2022 died from a cancer diagnosis [17]. This is also the case in Australia with 62% [18] and in the United States [19]. However more recently in the United States dementia has overtaken cancer as the primary diagnosis for those receiving hospice care [19]. In the UK, independent hospices are often the provider of palliative breathlessness groups which might explain the higher number of patients with COPD. This may be different in other international settings.

### Inequitable access to palliative rehabilitation

Inequitable access to palliative care has previously been criticised for patients from marginalised communities [20], especially those from black and ethnic minorities and lower socioeconomic backgrounds [21]. Some services in our survey reported an inability to offer translated information or interpretation services during rehabilitation for patients who do not have English as a first language. Language barriers have been reported in the literature as influential in poorer access to palliative care, poorer pain management, poorer support for caregivers, and poorer processing about treatment and prognosis [22]. The inability to offer translation or interpretation for patients who do not have English as a first language could therefore lead to poorer access to, and care from, community palliative rehabilitation services for this already marginalised population.

Inequity also exists in service delivery models with specialist palliative care offering more 1:1 and group services on the organisation’s site. Group based interventions have previously demonstrated positive psychological and physical outcomes in palliative care due to social support [23] therefore inability to access group-based services may lead to poorer outcomes. Finally, offering services on the organisational site may provide respite for carers, especially when transport is offered [24], however could provide a barrier to access for patients who are required to travel independently [24, 25].

### Integration with other services

The World Health Organisation states that rehabilitation should be integrated within palliative care [3], yet despite this only 29% of NHS generalist services felt integrated with specialist palliative care services. This integration was even less with other care settings. Integrating rehabilitation with other services enhances the knowledge of other professionals which can improve access for patients to rehabilitation [26]. It can also improve the quality, and efficiency of services while promoting holistic, person-centred care [3, 26].

### Workforce patterns

Our study findings support earlier research by Wosahlo & Maddocks’ [8] that physiotherapists were the most frequent AHPs in rehabilitation teams followed by occupational therapists. There was very little representative of other AHP professions. Recent recommendations in an EAPC white paper about frailty and ageing suggest that rehabilitation in palliative care should be integrative and multi-professional [27]. The World Health Organisation also highlights the importance of a multiprofessional workforce [3], however it is important to highlight the workforce differences between palliative rehabilitation and rehabilitative palliative care as mentioned in the introduction. This may be why other AHP professionals are not seen in rehabilitation teams.

This is the first study to identify the prevalence of clinical specialist physiotherapists and occupational therapists in the United Kingdom with independent hospices employing more than NHS rehabilitation teams. Workforce surveys in the United Kingdom and Australia have highlighted the need for specialist AHP roles embedded within specialist palliative care [28, 29]. There has also been a call for improved integration of rehabilitation into palliative care potentially leading to more specialist rehabilitation roles in this setting [3].

### Educational needs

Our study found that organisations providing community palliative rehabilitation reported that most physiotherapists and occupational therapists in both sectors wanted extra education and training to see patients with a palliative diagnosis. Other surveys in this area have mirrored our results demonstrating lower levels of confidence amongst all AHPs treating patients towards the end of life [30–32]. Better integration with specialist palliative care specialist services might improve support and confidence [33]. In this survey, however, independent hospice services reported good integration with specialist palliative care, but rehabilitation staff still sought further education or advice to treat this cohort of patients. Another solution might be more palliative and end of life care training at undergraduate level. Theory and practical education have been shown to improve confidence and self-efficacy in undergraduate nursing students [34, 35] indicating similar training may improve AHP confidence.

### Strengths and Limitations

This is the only study investigating UK models of community palliative rehabilitation delivery in both independent hospices and NHS Trusts. The survey findings suggest discrepancies between community palliative rehabilitation services and evidence-based practice, which, when addressed, could lead to enhanced service planning and clinical delivery.

Methodological strengths include using total sampling to minimise sampling error and ensure respondents were representative of the UK distribution of independent hospices and NHS trusts. Strategies such as ensuring unique IP addresses and CAPTCHA were used to minimise over-coverage error [36, 37]. Measurement error was reduced through pilot testing the questionnaire [37].

For organisations where a named rehabilitation lead could not be found, the survey was sent to a generic email address. This could have led to some under-coverage [36] as it is reliant on the organisation passing it on to the relevant individual. The response rate was low and some responses partially completed, which could have led to non-response bias [38] whereby organisations with no rehabilitation did not respond. Having said that, the response rate was similar to that of Wosahlo & Maddocks [8]. The survey was undertaken in the UK and therefore the results are not generalisable beyond the UK. Finally social desirability bias may exist due to senior managers completing the questionnaire [39], however respondents were encouraged to answer as accurately as possible and answers were able to be submitted anonymously.

### Clinical implications

The lack of AHP representation outside of physiotherapy and occupational therapy in community rehabilitation teams could mean additional referrals increasing waiting times for patients who already have limited time. This could lead to patients missing out on rehabilitation that could reduce pain, anxiety and depression and improve function. These will in-turn improve the quality of patients’ last year, months or days of life [40–42].

Poor integration between rehabilitation services and other primary, secondary, tertiary and specialist palliative care services might lead to missed or delayed referrals to rehabilitation teams. It is also attributed to poor quality of care and patient experience [43]. Improving integration between rehabilitation services and other primary and secondary care providers could allow for a sharing of knowledge between services about referral processes and patient care.

Clinicians working with palliative patients often seek further education or guidance to deliver palliative rehabilitation. Palliative care education delivered at undergraduate level or better integration with specialist palliative care might improve clinician confidence to treat palliative patients independently.

## Conclusion

The study highlights a significant gap between available community palliative rehabilitation services and evidence-based approaches documented in literature that could enhance outcomes for patients.

While physiotherapists and occupational therapists are commonly employed, other AHPs such as are frequently absent from rehabilitation teams. Independent hospices reported better integration with specialist palliative care and employed more clinical specialists. Significant equity concerns emerged for non-English speakers due to limited translation services and for patients who needed to be independent to access services. Even well-integrated settings reported needs for additional support to deliver rehabilitation to patients with a palliative diagnosis, indicating workforce development gaps.

To develop a model that enhances patient outcomes and optimises function and quality of life, key priorities include expanding multidisciplinary staffing, improving language accessibility, strengthening cross-sector integration, and increasing palliative care education.

## Supplementary Information

Supplementary Material 1.

## Data Availability

The datasets generated and analysed during the current study are available on the University of Sheffield data repository (ORDA) [https://orda.shef.ac.uk] (https://orda.shef.ac.uk). Please contact [rdm@sheffield.ac.uk] (mailto: rdm@sheffield.ac.uk) for access to this data.
