# Supplementary material for: What models of community palliative rehabilitation exist for adults in the United Kingdom? – a national cross-sectional survey
Source: BMC Palliat Care. 2026 Mar 13;25:100. doi: 10.1186/s12904-026-02027-x (PMC13085448; doi:10.1186/s12904-026-02027-x)
Supplement: Supplementary file 1 — Supplementary Material 1. [file 12904_2026_2027_MOESM1_ESM.docx]

Community Palliative Rehabilitation Questionnaire

Start of Block: Community Palliative Rehabilitation Survey

Q38 Welcome to the Community Palliative Rehabilitation Questionnaire

Thank you for agreeing to take part in this important questionnaire aiming to understand how palliative rehabilitation is delivered in the community in the United Kingdom. Please take the time to complete this questionnaire even if your service does not specialise in palliative rehabilitation, as this information is still relevant. 

The survey will take 12-15 minutes to complete and all responses will be anonymised. You will have the option at the end of the survey to enter your details to receive the overall results and to take part in further research. The anonymised results will be used to understand the models of care that palliative patients are receiving in the United Kingdom and will be published open access in a peer-reviewed journal. It is hoped that this information can be used alongside further research to develop guidelines for the delivery of palliative rehabilitation in the community.

Feel free to ask any questions about the project by contacting the project lead, Jane Manson, at jtmanson1@sheffield.ac.uk 

Please click the arrow to begin.

End of Block: Community Palliative Rehabilitation Survey

Start of Block: Your Organisation

Q1 Q1. Where is your organisation based?

- England (1)
- Northern Ireland (2)
- Scotland (3)
- Wales (4)
- Other - please state (5) __________________________________________________

Q2 Q2. Is your organisation?

- Hospice - charity funded (1)
- Hospice – Privately funded (2)
- NHS Hospice (3)
- NHS Palliative Care Unit (4)
- NHS Foundation Trust (5)
- Private Organisation (6)
- Other - please state (7) __________________________________________________

Q3 Q3 What is the best description of the area your organisation covers?

- Urban (1)
- Rural (2)
- Mixed urban and rural (3)
- Island (4)
- Other - please state (5) __________________________________________________

Q4 Q5 Please indicate the local population size that your organisation provides services for? If you are not sure, please give your best guess

- Under 50,000 (1)
- 50,000 – 100,000 (2)
- 100,000 – 250,000 (3)
- 250,000 – 500,000 (4)
- 500,000 - 750,000 (5)
- 750,000 - 1,000,000 (6)
- Over 1,000,000 (7)

End of Block: Your Organisation

Start of Block: The specific service you work in

Q5 Q6. Does the service that you work in deliver rehabilitation to palliative patients in the community (in their own homes, community centres, day rehabilitation, outpatients, or hybrid)?

- Yes (1)
- No (2)

Skip To: End of Survey If Q6. Does the service that you work in deliver rehabilitation to palliative patients in the commun... = No

End of Block: The specific service you work in

Start of Block: The specific service you work in

Q6 Q7. How many allied health professionals are employed by the community rehabilitation service you work in? (allied health professionals include art therapists, dietetics, drama therapists, lymphoedema specialists, music therapists, occupational therapists, orthotists, paramedics, physiotherapists, podiatrists, and speech and language therapists). If you are not sure please give your best guess

- 0 (1)
- Less than 1 FTE (2)
- 1 to 2 FTE (3)
- 3 to 5 FTE (4)
- 6 to 10 FTE (5)
- 11 to 15 FTE (6)
- 16 to 20 FTE (7)
- More than 20 FTE (8)

| Page Break |  |
| --- | --- |

Q7 Q8. Please indicate which allied health professional role(s) your service employs and how many, including any service level agreements in place. If you are not sure, please give your best guess.

|  | 0 (1) | Less than 1 FTE (2) | 1 to 2 FTE (3) | 3 to 5 FTE (4) | 6 to 10 FTE (5) | 11 to 15 FTE (6) | 16 to 20 FTE (7) | More than 20 FTE (8) |
| --- | --- | --- | --- | --- | --- | --- | --- | --- |
| Art Therapists (1) |  |  |  |  |  |  |  |  |
| Dieticians (2) |  |  |  |  |  |  |  |  |
| Drama Therapists (3) |  |  |  |  |  |  |  |  |
| Lymphoedema Specialists (4) |  |  |  |  |  |  |  |  |
| Music Therapists (5) |  |  |  |  |  |  |  |  |
| Occupational Therapists (6) |  |  |  |  |  |  |  |  |
| Orthotists (7) |  |  |  |  |  |  |  |  |
| Paramedics (8) |  |  |  |  |  |  |  |  |
| Physiotherapists (9) |  |  |  |  |  |  |  |  |
| Podiatrists (10) |  |  |  |  |  |  |  |  |
| Speech and Language Therapists (11) |  |  |  |  |  |  |  |  |
| Other (12) |  |  |  |  |  |  |  |  |

End of Block: The specific service you work in

Start of Block: The specific service you work in

Q9 Q9a. Do you have any palliative care clinical specialist physiotherapists (This means a clinician who has undergone specialist palliative care training and in the NHS is usually band 7 or above) within your service?

- Yes (1)
- No (2)

Display This Question:

If Q9a. Do you have any palliative care clinical specialist physiotherapists (This means a clinician... = Yes

Q11 Q9b. Please state how many palliative care clinical specialist physiotherapists work within your service

________________________________________________________________

Display This Question:

If Q9a. Do you have any palliative care clinical specialist physiotherapists (This means a clinician... = No

Q12 Q9b. Is there a service you can refer to for more specialist palliative physiotherapy support?

- Yes - please state who below (1) __________________________________________________
- No (2)

| Page Break |  |
| --- | --- |

Q13 Q10a. Do you have any palliative care clinical specialist occupational therapists (This means a clinician who has undergone specialist palliative care training and in the NHS is usually band 7 or above) within your service?

- Yes (1)
- No (2)

Display This Question:

If Q10a. Do you have any palliative care clinical specialist occupational therapists (This means a c... = Yes

Q14 Q10b. Please state how many palliative care clinical specialist occupational therapists work within your service

________________________________________________________________

Display This Question:

If Q10a. Do you have any palliative care clinical specialist occupational therapists (This means a c... = No

Q15 Q10b. Is there a service you can refer to for more specialist palliative occupational therapy support?

- Yes - please state who below (1) __________________________________________________
- No (2)

| Page Break |  |
| --- | --- |

Q16 Q11. Please indicate which profession takes the lead on palliative rehabilitation services.

- Physiotherapist or Occupational Therapist (1)
- Other allied health professional (2)
- Medical Staff (3)
- Nursing staff (4)
- Social work / counselling / psychologist / chaplain (5)
- Other - please state (6) __________________________________________________

End of Block: The specific service you work in

Start of Block: The palliative patients you see

Q17 Q12. What percentage of the patients seen in your community rehabilitation service are palliative (patients with advanced progressive illness)?

- Less than 10% (1)
- 10-25% (2)
- 25-50% (3)
- 50-75% (4)
- Over 75% (5)

Q18 Q13. Out of these palliative patients, how often does your community rehabilitation service see the following conditions?

|  | Very frequently/ daily (1) | Frequently/ weekly (2) | Occasionally/monthly (3) | Rarely/few times per year (4) | Never (5) |
| --- | --- | --- | --- | --- | --- |
| Cancer (1) |  |  |  |  |  |
| COPD (2) |  |  |  |  |  |
| Heart Failure (3) |  |  |  |  |  |
| Parkinson’s and related conditions (4) |  |  |  |  |  |
| Dementia (5) |  |  |  |  |  |
| Advanced Frailty (6) |  |  |  |  |  |
| End-stage kidney disease (7) |  |  |  |  |  |
| End stage liver disease (8) |  |  |  |  |  |
| Motor Neurone Disease (9) |  |  |  |  |  |
| Other progressive neurological conditions (10) |  |  |  |  |  |
| Other - Please state (11) |  |  |  |  |  |

End of Block: The palliative patients you see

Start of Block: The palliative patients you see

Q19 Q14. Where do referrals for palliative patients into your service come from?

|  | Very frequently/daily (1) | Frequently/ weekly (2) | Occasionally/monthly (3) | Rarely/few times per year (4) | Never (5) |
| --- | --- | --- | --- | --- | --- |
| Hospital discharge (1) |  |  |  |  |  |
| General practitioner (GP) (2) |  |  |  |  |  |
| District nurse (3) |  |  |  |  |  |
| Specialist outpatient clinic (oncology, respiratory, cardiac, neurology etc) (4) |  |  |  |  |  |
| Specialist nurse (specialist tumour nurse, heart failure nurse, COPD nurse) (5) |  |  |  |  |  |
| Hospice/palliative care service (6) |  |  |  |  |  |
| Generalist therapy service (7) |  |  |  |  |  |
| Self-referral (8) |  |  |  |  |  |
| Other - please state (9) |  |  |  |  |  |

End of Block: The palliative patients you see

Start of Block: The service you provide to palliative patients

Q20 Q15. Are there criteria for patients to access your service?

- Yes (1)
- No (2)

Skip To: End of Block If Q15. Are there criteria for patients to access your service? = No

Display This Question:

If Q15. Are there criteria for patients to access your service? = Yes

Q21 Q15b. Please indicate which criteria these are (tick all that apply)

- Need own transport (1)
- Need to be able to toilet self (2)
- Need to be mobile (with/without aid) (3)
- Must be referred by Healthcare professional referral (4)
- Have a physical problem or need (5)
- Have a problem caused by palliative illness (e.g. pain, breathlessness, anxiety etc) (6)
- Have a certain prognosis (i.e. less than a year to live) (7)
- Be able to access appointments virtually (8)
- Have a carer present (9)
- Be able to follow simple instructions (10)
- Be able to follow instructions in English (11)
- Other, please state (12) __________________________________________________

End of Block: The service you provide to palliative patients

Start of Block: The service you provide to palliative patients

Q22 Q16. Does your community rehabilitation service offer the following models to patients?

|  | Yes (1) | No (10) |
| --- | --- | --- |
| In the person’s own home (1) |  |  |
| One to one not on organisation site (NHS/Hospice etc) (2) |  |  |
| Group not on organisation site (NHS/Hospice etc) (3) |  |  |
| One to one on organisation site (NHS/Hospice etc) (4) |  |  |
| Group on organisation site (NHS/Hospice etc) (5) |  |  |
| Telerehabilitation (rehabilitation delivered remotely via telephone, video or other assisted devices) (6) |  |  |
| Other - please state (7) |  |  |

Display This Question:

If Q16. Does your community rehabilitation service offer the following models to patients? = Yes

Q23 Q17. In your experience of providing telerehabilitation (rehabilitation delivered remotely via telephone, video or other assisted devices) have you encountered any of the following? (please tick all that apply)

- Improved access to therapy service where otherwise patients wouldn’t have been able to access rehabilitation (1)
- Reduced access to the therapy service due to no access to or reluctance to use technology or internet-based services (2)
- Concerns from staff-members regarding safety of telerehabilitation (3)
- Concerns from patients regarding safety of telerehabilitation (4)
- Positive feedback from staff-members regarding the telerehabilitation service provided (5)
- Positive feedback from patients regarding the telerehabilitation service provided (6)
- Negative feedback from patients regarding the telerehabilitation service provided (7)
- Issues around integration with existing electronic medical records (8)

Display This Question:

If Q16. Does your community rehabilitation service offer the following models to patients? = Telerehabilitation (rehabilitation delivered remotely via telephone, video or other assisted devices) [ No ]

Q24 Q17. Which of the following reasons do you think are a barrier to providing telerehabilitation? (please tick all that apply)

- Lack of technology available at my organisation (1)
- Concern about reduced rehabilitation due to no access to or reluctance to use technology or internet-based services (2)
- Concern that you can’t provide as good psychological assessment and intervention using telerehabilitation (3)
- Concern that you can’t provide as good physical assessment and intervention using telerehabilitation (4)
- Concern that patients won’t engage with telerehabilitation (5)
- Lack of staff confidence in telerehabilitation (6)
- Concern for patient safety when providing telerehabilitation (7)
- Other - please state (8)

End of Block: The service you provide to palliative patients

Start of Block: The service you provide to palliative patients

Q25 Q18. Please indicate what types of rehabilitation interventions are offered by your service

|  | Daily /very frequently (1) | Weekly /frequently (2) | Monthly /regularly (3) | Six monthly /infrequently (4) | Yearly /rarely (5) | Never (6) |
| --- | --- | --- | --- | --- | --- | --- |
| Anxiety/stress management (1) |  |  |  |  |  |  |
| Breathlessness management (2) |  |  |  |  |  |  |
| Cognitive/memory/perceptual interventions (3) |  |  |  |  |  |  |
| Creative arts/crafts (4) |  |  |  |  |  |  |
| Energy conservation (5) |  |  |  |  |  |  |
| Exercise (6) |  |  |  |  |  |  |
| Falls prevention (7) |  |  |  |  |  |  |
| Fatigue management (8) |  |  |  |  |  |  |
| Hydrotherapy/swimming (9) |  |  |  |  |  |  |
| Minor adaptations and equipment provision (10) |  |  |  |  |  |  |
| Non-pharmacological pain management (11) |  |  |  |  |  |  |
| Positioning and manual handling (12) |  |  |  |  |  |  |
| Reminiscence (13) |  |  |  |  |  |  |
| Other (please state) (14) |  |  |  |  |  |  |

End of Block: The service you provide to palliative patients

Start of Block: The service you provide to palliative patients

Q26 Q19. Please give your answer to the following questions about the holistic nature of your service

|  | Always (1) | Usually (2) | Sometimes (3) | Rarely (4) | Never (5) | Don't know (6) |
| --- | --- | --- | --- | --- | --- | --- |
| Is goal setting used with patients? (1) |  |  |  |  |  |  |
| Are you able to offer rehabilitation at a time which suits the patient? (2) |  |  |  |  |  |  |
| Can you offer different formats or locations to meet someone’s needs (e.g. home-based, outpatient, one-to-one, group-based, virtual) (3) |  |  |  |  |  |  |
| Do you offer information in languages other than English? (4) |  |  |  |  |  |  |
| Are you able to offer interpreter services for patients who do not have English as a first language? (5) |  |  |  |  |  |  |
| Are the patients that access your service representative of your organisations' catchment area? (7) |  |  |  |  |  |  |
| Do therapists working in your service report they need further education or advice to provide rehabilitation to palliative patients? (6) |  |  |  |  |  |  |

Display This Question:

If Q19. Please give your answer to the following questions about the holistic nature of your service = Do therapists working in your service report they need further education or advice to provide rehabilitation to palliative patients? [ Always ]

And Q19. Please give your answer to the following questions about the holistic nature of your service = Do therapists working in your service report they need further education or advice to provide rehabilitation to palliative patients? [ Usually ]

And Q19. Please give your answer to the following questions about the holistic nature of your service = Do therapists working in your service report they need further education or advice to provide rehabilitation to palliative patients? [ Sometimes ]

And Q19. Please give your answer to the following questions about the holistic nature of your service = Do therapists working in your service report they need further education or advice to provide rehabilitation to palliative patients? [ Rarely ]

Q38 Q19b To the best of your knowledge, where do therapists usually get that education or advice?

|  | Yes (1) | No (2) | Don't know (3) |
| --- | --- | --- | --- |
| Physiotherapist or occupational therapist employed by the same organisation (1) |  |  |  |
| Physiotherapist or occupational therapist employed by a different organisation (2) |  |  |  |
| Clinical Specialist physiotherapist or occupational therapist employed by same organisation (3) |  |  |  |
| Clinical Specialist physiotherapist or occupational therapist employed by a different organisation (4) |  |  |  |
| Inservice training in own service (5) |  |  |  |
| Training provided by own organisation (6) |  |  |  |
| External training to own organisation (7) |  |  |  |
| Chartered Society of Physiotherapy or Royal College of Occupational Therapy (8) |  |  |  |
| Special interest group (9) |  |  |  |
| Research articles (10) |  |  |  |
| Social media (11) |  |  |  |
| Other - please state (12) |  |  |  |

End of Block: The service you provide to palliative patients

Start of Block: Your service and integration

Q29 Q20. Please give details about how you work alongside other services

|  | Own service (1) | Cross-organisational service delivery e.g. joint funded AHP posts, joint group programmes (2) | Joint clinical meetings e.g. MDT (3) | Liaison only e.g. telephone and letters (4) | No collaboration (5) | Not applicable (6) |
| --- | --- | --- | --- | --- | --- | --- |
| Community NHS (1) |  |  |  |  |  |  |
| Hospice (2) |  |  |  |  |  |  |
| Private health / rehabilitation service (3) |  |  |  |  |  |  |
| Social Care (4) |  |  |  |  |  |  |
| Other voluntary organisation (5) |  |  |  |  |  |  |
| Other (6) |  |  |  |  |  |  |

Q30 Q21. Please give your answer to the following questions about the integration of your service.

|  | Yes (1) | To some extent (2) | No (3) |
| --- | --- | --- | --- |
| Do you think that your service is well integrated with specialist palliative care services such as nurses, doctors and other specialist clinicians? (1) |  |  |  |
| Do you think that your service is well integrated with other primary care services such as district nurses, GPs and other community healthcare professionals? (2) |  |  |  |
| Do you think that your service is well integrated with secondary care services (3) |  |  |  |
| Do you think that your service is well integrated with the third sector (community groups, voluntary organisations, faith and equalities groups, charities, housing associations) (4) |  |  |  |
| Do you think other clinicians who could refer into your service understand the service that you can provide to palliative patients? (5) |  |  |  |
| Do you feel that palliative patients are referred early enough into your service for you to make a difference? (6) |  |  |  |

End of Block: Your service and integration

Start of Block: About you

Q31 Q22. What is your profession?

- Physiotherapist - clinician (1)
- Occupational Therapist - clinican (2)
- Physiotherapist - service lead (3)
- Occupational therapist - service lead (4)
- Nurse - clinician (5)
- Nurse - service lead (6)
- Other allied health professional - clinican (7)
- Other allied health professional - service lead (8)
- Service lead - other (9)
- Other - please state (10) __________________________________________________

Q32 Q23. Please give your NHS grade (or equivalent) if known

- NHS band 5 or equivalent (1)
- NHS band 6 or equivalent (2)
- NHS band 7 or equivalent (3)
- NHS band 8a or equivalent (4)
- NHS band 8b or higher or equivalent (5)
- Not sure (6)

End of Block: About you

Start of Block: Any other comments

Q33 Q24. It would be extremely beneficial to the study to understand the things that you feel that your community rehabilitation service does well and could do better. This sharing of knowledge may help to shape services in the future. Please write any thoughts you have in the box below.

________________________________________________________________

| Page Break |  |
| --- | --- |

Q34 Q25. As mentioned in the invitation e-mail, the next part of this research is to understand services in more detail. Would you be willing to be contacted to discuss this further for your service?

- Yes (1)
- No (2)

Display This Question:

If Q25. As mentioned in the invitation e-mail, the next part of this research is to understand servi... = Yes

Q35 Q25b. Please provide your name and email if willing to be contacted

- Name (1) __________________________________________________
- Email address (2) __________________________________________________

Q35 Would you like to receive the results of this research once completed?

- Yes (1)
- No (2)

Q36 Please provide your name and email to send the results to

- Name (1) __________________________________________________
- Email address (2) __________________________________________________

End of Block: Any other comments
